# Combined effect of dose gradient and rotational error on prescribed dose coverage for single isocenter multiple brain metastases in frameless stereotactic radiotherapy

**DOI:** 10.1186/s13014-021-01893-4

**Published:** 2021-08-31

**Authors:** Jai-Woong Yoon, Soah Park, Kwang-Ho Cheong, Sei-Kwon Kang, Tae Jin Han

**Affiliations:** 1 Department of Radiation Oncology, Dongtan Sacred Heart Hospital, Hwaseong, Korea; 2 Department of Radiation Oncology, Kangnam Sacred Heart Hospital, Hallym University College of Medicine, Seoul, Korea; 3 Department of Radiation Oncology, Hallym University Sacred Heart Hospital, Hallym University College of Medicine, Anyang, Korea; 4 Department of Radiation Oncology, Kangdong Sacred Heart Hospital, Hallym University College of Medicine, Seoul, Korea

**Keywords:** Frameless stereotactic radiotherapy, Single isocenter multiple brain metastases, Rotational error, Dose gradient, Dose coverage

## Abstract

**Background:**

To evaluate the combined effect of rotational error and dose gradient on target dose coverage in frameless stereotactic radiotherapy.

**Methods:**

Three spherical targets of different diameters (1, 1.5, and 2 cm) were drawn and placed equidistantly on the same axial brain computed tomography (CT) images. To test the different isocenter-target distances, 2.5- and 5-cm configurations were prepared. Volumetric modulated arc therapy plans were created for different dose gradients from the target, in which the dose gradients were modified using the maximum dose inside the target. To simulate the rotational error, CT images and targets were rotated in two ways by 0.5°, 1°, and 2°, in which one rotation was in the axial plane and the other was in three dimensions. The initial optimized plan parameters were copied to the rotated CT sets, and the doses were recalculated. The coverage degradation after rotation was analyzed according to the target dislocation and 12-Gy volume.

**Results:**

A shallower dose gradient reduced the loss of target coverage under target dislocation, and the effect was clearer for small targets. For example, the coverage of the 1-cm target under 1-mm dislocation increased from 93 to 95% by increasing the Paddick gradient index from 5.0 to 7.9. At the same time, the widely accepted necrosis indicator, the 12-Gy volume, increased from 1.2 to 3.5 cm^3^, which remained in the tolerable range. From the differential dose volume histogram (DVH) analysis, the shallower dose gradient ensured that the dose-deficient under-covered target volume received a higher dose similar to that in the prescription.

**Conclusions:**

For frameless stereotactic brain radiotherapy, the gradient, alongside the margin addition, can be adjusted as an ancillary parameter for small targets to increase target coverage or at least limit coverage reduction in conditions with probable positioning error.

## Introduction

Linear accelerators have been widely employed for stereotactic radiosurgery (SRS) and fractionated stereotactic radiation treatment (FSRT). With the rapid progress in image-guided radiotherapy (IGRT), the framed era has evolved to noninvasive frameless fixation using a mask [1–5]. Because SRS/FSRT delivers an unusually high dose to the target in a single or few fractions, high targeting accuracy is necessary for complete tumor eradication and normal brain protection. In addition to being able to provide IGRT, a couch with six degrees of freedom for position correction is considered an essential accessory for radiosurgery [6–8].

The initial setup error in patient preparation is routinely verified using image guidance, such as cone beam computed tomography (CBCT) or stereoscopic X-ray imaging. However, with concerns regarding probable intrafractional error during treatment, several groups have studied target positional accuracy and its dosimetric effect [9–12]. Some researchers have simulated rotational and translation errors for multiple targets and found that the target size and distance between the isocenter and targets are important for maintaining the desired target coverage.

However, we note that target coverage may be vulnerable to a steeper dose gradient for the same displacement error of the target. When the dose gradient is steeper, for a given positional error, the dose coverage of the target is expected to be lower than with a shallower gradient.

In this study, we assess the dosimetric effect of rotational error on single-isocenter multi-target coverage with various dose gradients for noninvasive mask-based SRS and FSRT.

## Methods

Multiple metastases were simulated on a brain CT image set with a 1-mm slice thickness. Three nearly spherical targets with diameters of 1 cm (“T1cm,” volume of 0.6 cm^3^), 1.5 cm (“T1.5cm,” 1.9 cm^3^), and 2 cm (“T2cm,” 4.4 cm^3^) were drawn and located equidistantly in the axial plane along a circle with a radius of 2.5 cm. The center of the circle was set as the beam isocenter (“iso-radius 2.5 cm”). To investigate scenarios with a larger iso-radius, similar artificial targets were prepared for a circle with a 5-cm radius (“iso-radius 5 cm”).

**Fig. 1:**
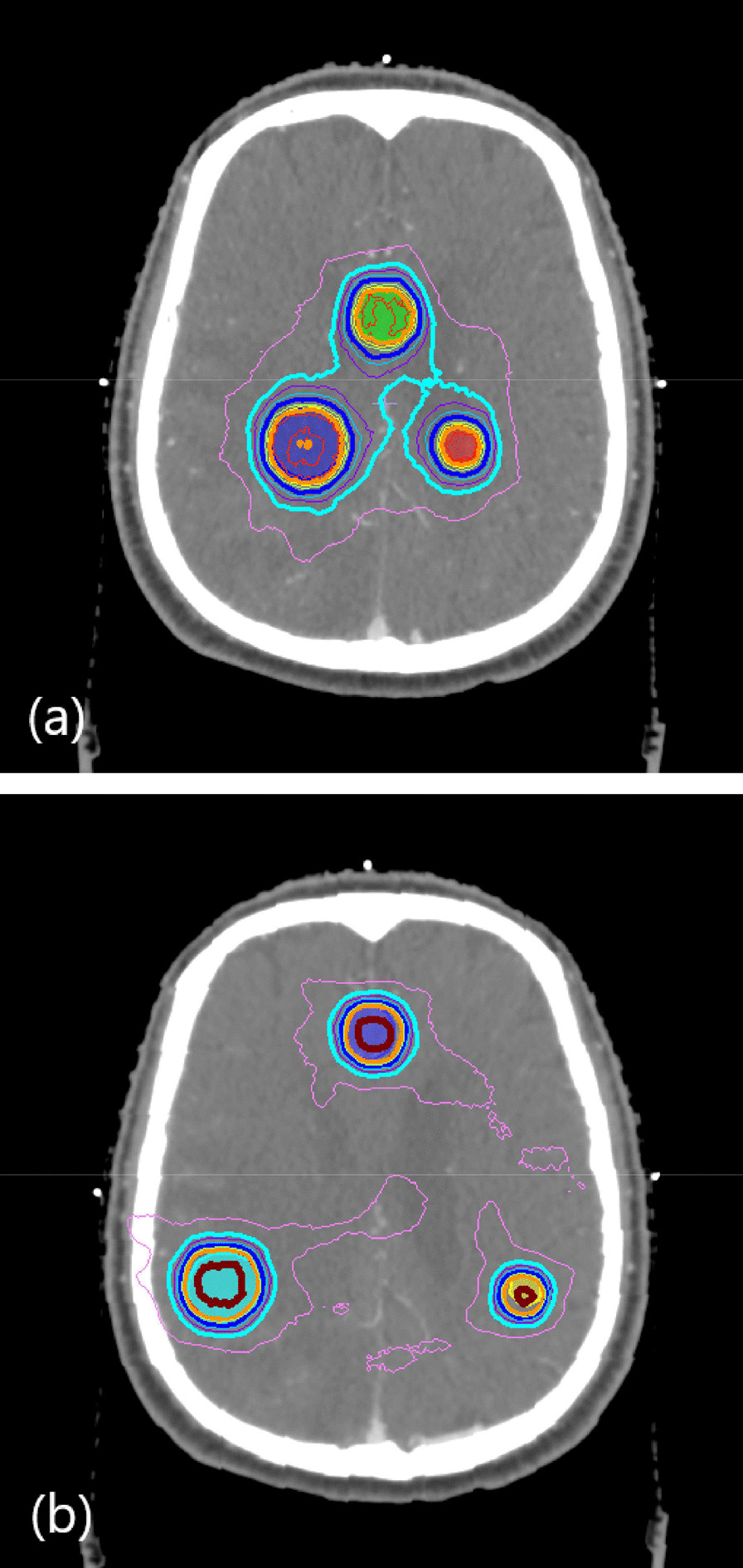
Two example plan cases of r2.5D110 and 2° rotated 3Dr5D150. **a** A plan case in which the distance from the isocenter to targets (iso-radius) is 2.5 cm with a nominal 110% maximum dose allowed inside the target. No rotation. **b** Three-dimensionally 2° rotated case with 5-cm iso-radius with 150% maximum dose inside the target

Plans were created in Monaco 5.0, with agilant MLC of VersaHD of Elekta, using three non-coplanar VMAT beams with couch angles of 0° (arc range: 360°), 45° (arc: 10°–170°), and 90° (arc: 10°–170°). The prescription was 18 Gy to 97% of the target volume, and the dose calculation grid was 1 mm. With the aim of varying the degree of dose gradient around the target, the dose maximum inside the target was allowed during optimization to be approximately 110%, 130%, and 150% of the prescription dose (19.8, 23.4, and 27 Gy, respectively). Appropriate dose control around the target was also applied with respect to gradient variation. Naming is helpful for distinguishing between plans; for example, r2.5D110 is the plan with an iso-radius of 2.5 cm and permitted maximum dose of 110%, and has three targets, r2.5T1cm, r2.5T1.5 cm, and r2.5T2cm (Fig. 1).

To simulate the effect of target displacement error, the CT image was rotated by 0.5°, 1.0°, and 2.0° about the isocenter as the rotation center. Rotation was performed in the axial plane and in three dimensions (3D) around the x-, y-, and z-axes. The original CT set and each rotated set were rigidly fused in Monaco, and the original targets were copied to the rotated CT set. The variations in copied target volumes were less than 3% for the 1-cm target and less than 1.0% for the 1.5-cm and 2-cm targets. The optimized plan parameters for each gradient were copied to every rotated image, and doses were recalculated. Therefore, including the original six unrotated plans, study plans were prepared for each combination of two rotation methods (axial and 3D), three different values of gradient steepness, three different rotation degrees, and two inter-target distances, resulting in a total of 42 plans. The Paddick gradient index (GI) was used for gradient evaluation around the tumor, which is a metric of the dose steepness around the target [13]. The GI is equal to V_50%_/V_100%_, i.e., the ratio of the volume receiving half the prescription dose (V_50%_) to the prescription dose volume (V_100%_).

## Results

**Fig. 2:**
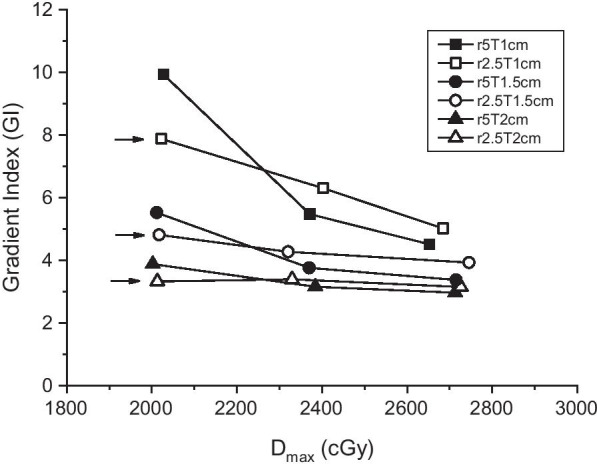
Paddick gradient index (GI) versus the permitted maximum dose inside the target. GI is lower (steeper gradient) as the maximum dose is higher. Arrowed GI values are those of r2.5D110 and calculated using 11-Gy volumes, not 9 Gy; therefore, they are underestimated

Plans with varying dose gradients around the target could be created by allowing different maximum doses inside the target and surrounding ring-shaped regions. Figure 2 shows that the GI was smaller (steeper gradient) for a higher dose maximum. The GI is calculated using the volumes of the target dose and half target dose, 18 and 9 Gy, respectively. However, for the case of r2.5D110 shown in Fig. 2, the 9-Gy volumes were not separated for each target because of the low dose bridges between targets. Therefore, a volume of 11-Gy was used in this case, resulting in lower GI values than expected. Gradient control is prominent in small targets.

**Fig. 3:**
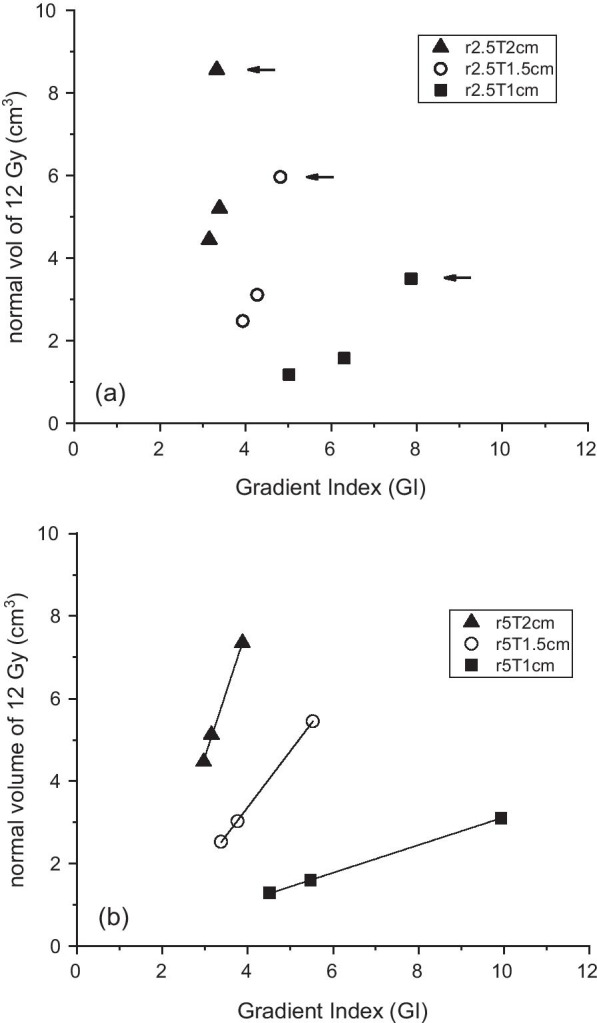
Normal brain volume irradiated with 12 Gy versus GI. **a** Results with iso-radius of 2.5 cm and **b** 5 cm with a linear fit. Arrowed GI values are underestimated as explained before, and expected to be similar to those obtained with an iso-radius of 5 cm. The steeper gradient (smaller GI) leads to a smaller volume irradiated with 12 Gy, and the linear relationship can be seen in (**b**)

The brain tissue volume irradiated with 12 Gy after subtracting the target (V12) was considered the normal brain necrosis indicator. For smaller GI values, V12 was smaller, and the reduction was larger for larger targets, as shown in Fig. 3, in which a linear relationship can be observed. In the case of a 1-cm target with 5 cm iso-radius (r5T1cm), the volume fell by 1.8 cm^3^ when the GI value changed from 9.9 (D110) to 4.5 (D150). The 2-cm target (r5T2cm) showed a 2.9 cm^3^ reduction from 3.9 (D110) to 3.0 (D150).

**Fig. 4:**
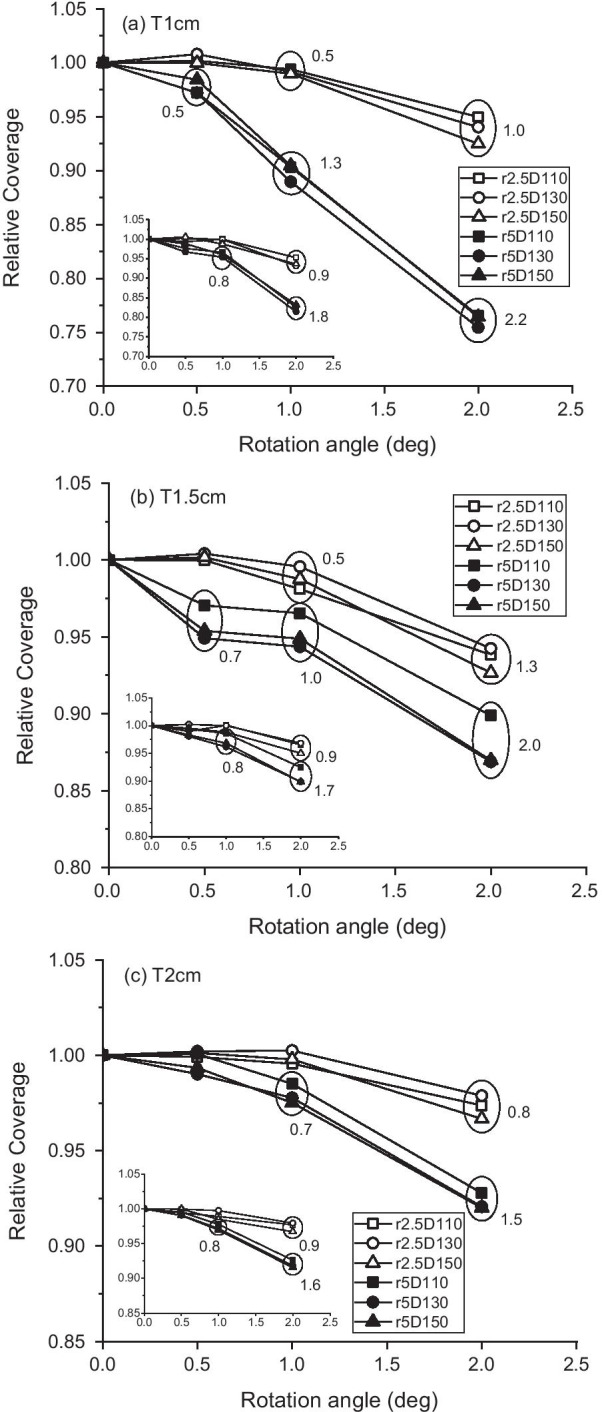
Relative target coverage vs. rotational error for 3D rotation and axial rotation (inset). Target coverage was normalized to those of rotation-free cases. **a** 1-cm targets for different values of gradient steepness and iso-radius, **b** 1.5-cm targets, and **c** 2-cm targets. Smaller targets receive less coverage following rotation. Numbers next to data points are displacement values in mm

The target dose coverage changed with rotational error, as shown in Fig. 4, in which the 3D rotation results are displayed with those for axial rotation in insets. The coverage was normalized to that of the unrotated case to show the variation by rotation only. The coverage varies in a complex manner with iso-radius, target size, dose gradients, and rotation method (axial or 3D). The variations in the iso-radius and rotation methods arise from the different displacements of the target from the original position. In the case of axial rotation, the displacement is uniform because all targets are located equidistantly from the rotation center (also beam isocenter) for each iso-radius of 2.5 and 5 cm. However, in the case of 3D rotation, the distance from the rotation axis, x, y, and z, differ for each target and cause different dislocations, even for the same 3D rotation. For example, under the same 3D rotation of 2° with iso-radius of 5 cm (r5rot2), the dislocations were 2.2 mm, 2.0 mm, and 1.5 mm for T1cm, T1.5 cm, and T2cm, respectively. The displacement of each target from the respective original position was calculated using the center position of each target before and after rotation, as shown in Table 1. For a better understanding, the displacement value in mm for parts of the targets is shown in Fig. 4.

**Table 1:** Displacement of targets from their original positions after rotation in mm

	T1cm	T1.5 cm	T2cm
3Dr2.5rot0.5	0.3	0.4	0.2
3Dr2.5rot1	0.5	0.5	0.3
3Dr2.5rot2	1.0	1.3	0.8
3Dr5rot0.5	0.5	0.7	0.3
3Dr5rot1	1.3	1.0	0.7
3Dr5rot2	2.2	2.0	1.5
AXr2.5rot0.5	0.2	0.4	0.3
AXr2.5rot1	0.4	0.4	0.4
AXr2.5rot2	0.9	0.9	0.9
AXr5rot0.5	0.4	0.5	0.4
AXr5rot1	0.8	0.8	0.8
AXr5rot2	1.8	1.7	1.6

3D for three-dimensional rotation and AX for axial rotation

Overall, although the coverage shows variations in target size and gradient steepness based on the maximum dose, the larger displacement error induced less coverage for targets of all sizes. For all targets with dislocation less than 0.8 mm, the target dose coverage was greater than 95%. The worst coverage (75%) was seen for the 1-cm target with an iso-radius of 5 cm undergone 3D rotation of 2° (2.2-mm dislocation). Although the effects were not dramatic, the steeper gradient negatively affected the coverage for all targets.

**Fig. 5:**
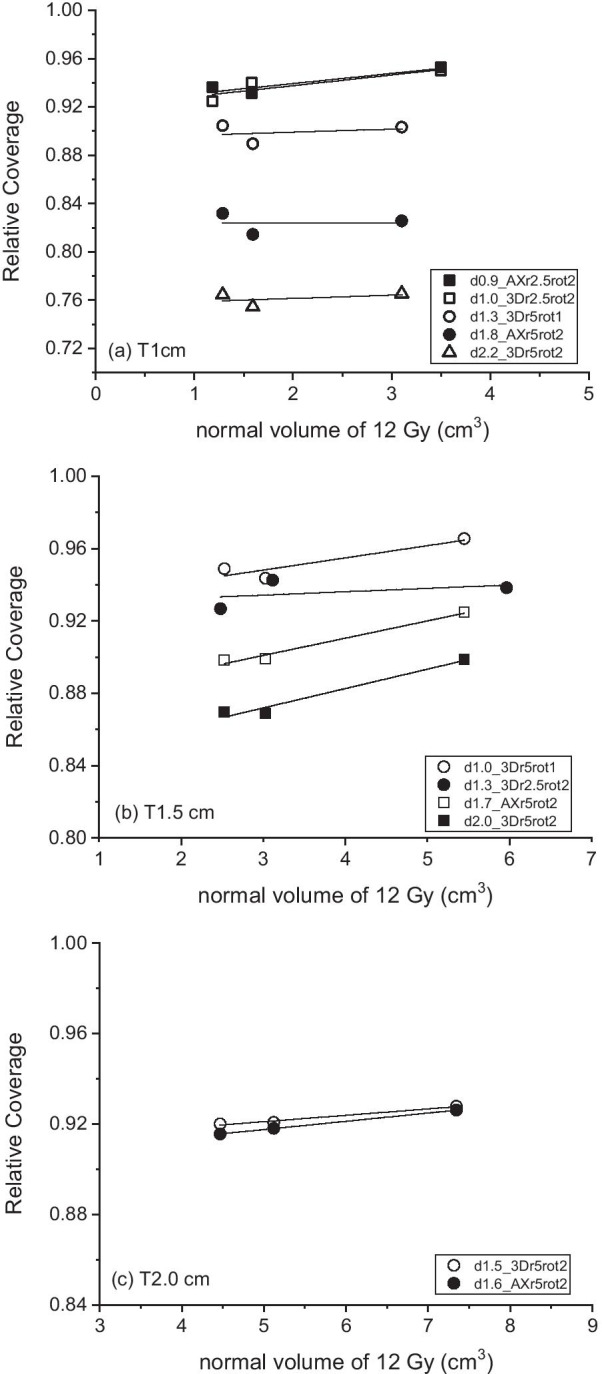
Relative target coverage for normal brain volume of 12 Gy. Data for target displacement larger than 0.9 mm are plotted. **a** 1-cm targets, **b** 1.5-cm targets, and **c** 2-cm targets. Lines represent a linear fit. Legend: d0.9_AXr2.5rot2 represents an iso-radius of 2.5 cm with axial rotation (AX) of 2° resulting in a dislocation of 0.9 mm from the original position. 3D stands for three-dimensional rotation

The cases with positional error greater than 0.9 mm are shown in Fig. 5, in which coverage was associated with a normal brain volume of 12 Gy (V12) corresponding to gradient steepness. The coverage clearly decreased more for larger displacement errors, and similar displacement resulted in similar coverage outcomes. A larger volume of 12 Gy is caused by a shallower gradient which was controlled by a lower maximum dose inside the target. For all three targets, a linear relationship can be observed between coverage and V12. For T1cm with a dislocation of 0.9 mm and 1.0 mm, the mean coverage increases from 93.1 to 95.1% with an increase of V12 from 1.2 cm^3^ (D150, GI of 5.0) to 3.5 cm^3^ (D110, GI of 7.9). Larger shifts with coverage of less than 90% did not guarantee a similar increase with an increase of V12. In the case of T1.5 cm, the increase in coverage was similar that an increase of approximately 2% can be seen for a change in V12 of 2.9 cm^3^. In the same way, the mean coverage of T2cm under dislocations of 1.5 mm and 1.6 mm changed from 91.8 to 92.7% for a V12 from 4.5 to 7.3 cm^3^.

**Fig. 6:**
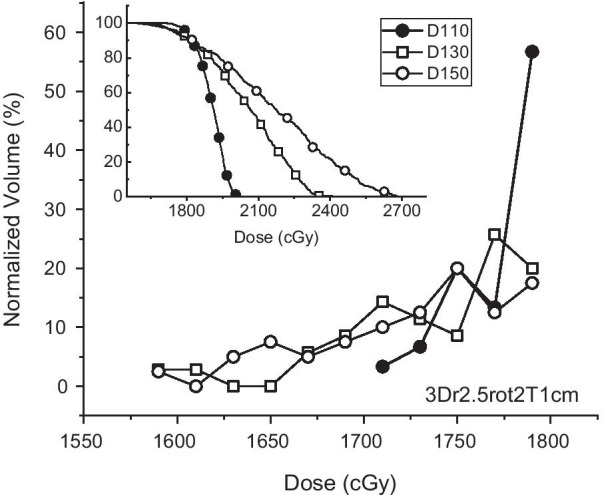
Differential DVHs under 18 Gy for different dose gradients for 1-cm targets with an iso-radius 2.5 cm rotated three dimensionally by 2° (3Dr2.5rot2). Inset are the corresponding cumulative DVHs after 15 Gy

Figure 6 shows an example of differential and cumulative dose volume histograms (dDVH and cDVH, respectively) for a T1cm with an iso-radius of 2.5 cm undergoing a 3D rotation of 2° (3Dr2.5rot2). The cDVH shows longer tails and a higher maximum dose for the target under a steeper gradient. In addition, even with similar target coverage of the prescription dose, the dose-volume patterns below the prescription dose (18 Gy) are different from each other, which can be clearly identified using dDVH. For dDVH, only the volume of the target receiving less than 18 Gy was normalized using a bin width of 20 cGy. In the case of D110, 56.7% of the dose-deficient volume received 17.8–18 Gy and 13.3% received 17.6–17.8 Gy. For D130 and D150, 20.0/25.7 and 17.5/12.5% received each of these doses, respectively. The dose gradient affects the dose-volume characteristics below the prescribed dose such that shallower dose steepness guarantees less deviation of the target volume from the prescription dose.

## Discussion

### Target dislocation and dose coverage

The study of positional error and its effect on the target coverage in frameless mask fixation has been performed by several groups. Tarnavski et al. assessed the fixation capability of the thermoplastic mask and observed 2- and 3-mm location errors in 6 and 2% of all beams, respec tively. A rotational error greater than 2° was also detected in 2.7% [14]. According to Gevaert et al., when using the BrainLAB mask (BrainLAB AG, Feldkirchen, Germany), the mean intrafractional shift was 0.58 mm (SD, 0.42 mm) and the rotational error was less than 0.03° (SD, 0.33°) for each direction. However, 9 out of the 66 targets had > 1 mm intrafractional motion and 18 out of the 66 lesions had rotational motion > 0.5°, which were their action levels [6]. In a simulation of rotational error that rotated the reference dose distribution around each of the three axes, Prentou et al. showed that the lesion size and distance from the target to the isocenter were important; the target coverage of 2.5 cm^3^ dropped to 84% for 1° error, and 62% for 2° error [12]. Smaller targets < 1 cm^3^ were more prone to coverage loss due to rotational error.

Our study differs in that the dose fall-off around the target was considered along with the positional error. The simulation results could be analyzed consistently based on the target dislocation, irrespective of whether the rotation method is axial or three-dimensional. The combination of the rotational error and gradient variation in our study revealed that decreasing gradient steepness increases the target coverage for the same positional error. For targets of 1, 1.5, and 2 cm in size, a dislocation of 0.8 mm had little effect on coverage, maintaining more than 95% of the original plan for all dose gradients studied. From the analysis of the cases with > 0.9 mm dislocation, coverage increased by 2% by changing the gradient via the maximum dose from D150 to D110 for T1cm and T1.5 cm. The increase in the T2cm was approximately 1%. Although a dramatic increase was not produced, decreasing the gradient had a positive effect for all target sizes under study.

Simulation accuracy needs to be mentioned. The change in coverage could be partly affected by the calculation uncertainty of the target volume after rotation, especially for small targets such as T1cm. However, because the volume change of the copied target was less than 3%, the large variation in coverage from rotation (i.e., dislocation) is reliable. In addition, this change is not related to the coverage variation for dose steepness because when we compared the coverage for dose steepness at fixed rotation, the same copied target was used with varying dose gradients. In addition, the rotation of the CT image could alter the pixel entity inside the dose grid. However, we adopted a 1-mm resolution for the dose grid, which was the smallest in Monaco. Moreover, brain tissue could be regarded as homogeneous. Therefore, we conclude that the simulation uncertainty is acceptable.

Another advantage of decreasing the gradient is the enhancement of the dose characteristics of the undercovered target volume. From the dDVH, even with similar target coverage, a shallower gradient reduced the dose deficiency in the volume covered by less than the prescription dose. A shallower gradient can be regarded as limiting dose deficiency.

### Strategies to tackle target dislocation and dose deficit

A few strategies can be considered to handle target dislocation and its negative effects. The first is head immobilization. For example, the fixation ability of a mask with a patient-specific dental bite block attached to the frame was estimated to be comparable to that of the invasive frame method [3, 15]. However, a null result was also obtained using the bite-block system by Ohira et al. [16] which implies that the accuracy evaluation at each institution is required before use.

Repetitive imaging during treatment to relocate the target when necessary is an active approach. Unlike CBCT, which is capable of imaging at zero couch angles only, stereoscopic x-ray imaging (ExacTrac, BrainLab AG, Germany) and surface guided imaging (SGRT) with no radiation are options to be considered [6, 7, 17, 18]. According to Barnes et al., 42% of fractions required repositioning when intrafractionally monitored using stereoscopic X-ray imaging because of motion greater than 0.7 mm and 0.7°, which was their action level [7]. However, the prolonged treatment time required for repetitive imaging and position correction cannot be neglected.

Target margin is another approach. In a rotational error simulation, Peng et al. advised a 3-mm CTV-to-PTV margin to cover the no less than 95% of the CTV for most target cases with ≤ 3° rotational error [9]. Usually, a 1–2-mm margin to the CTV was suggested for frameless SRS and FSRT [5–8, 19]. Recently, a method to create an optimal margin robust to positional errors has also been reported [20]. However, a higher margin indicates more normal brain tissue irradiation. For a 1-cm target, the addition of 1-mm margin increases the target volume of 0.52 to 0.90 cm^3^. In the case of a 1.5-cm target, the volume changes from 1.77 to 2.57 cm^3^. Most studies on target motion have assumed that the movement occurred around the beam isocenter which was exactly aligned during the patient setup. However, it is not possible to guarantee that the motion occurs just around the isocenter. In addition, contrary to the frame-based, frameless setup has an increased possibility of involuntary movement. The PTV margin cannot ensure sufficient coverage for all possible movements.

Our study showed that the dose fall-off around the target can be used in addition to all the above-mentioned strategies. In our simulation study, a shallower dose gradient decreases the reduction in target coverage caused by positional error, and the effect is more prominent for smaller targets. In the case of the 1-cm target, the coverage following 1.0-mm dislocation increased from 93.1 to 95.1%. Likewise, it increased from 91.8 to 92.7% for T2cm under a dislocation of 1.5 mm. However, at the same time, a shallower dose gradient increases the net volume of tissue exposed to 12-Gy irradiation, which is recommended to be < 10 cm^3^ or < 7.9 cm^3^ to limit probable normal tissue necrosis [21, 22]. In Fig. 5, 1- and 1.5-cm targets with any gradient and 2-cm targets with a GI < 3.2 (D130) satisfied the volume criteria. Of course, the increase in 12-Gy volume should be assessed with caution for the number of targets and prescription dose. Lowering the gradient by allowing the maximum dose to be 130% rather than 150% [5] is a good balance for small targets ≤ 1 cm in size to protect against probable dislocation error. When critical organs are near the target, the dose gradient should be steep as usual.

This study is not based on real tumor cases in various environments, and the simulation of motion error has limitations. However, the combined results from axial and three-dimensional rotations present ideas on how the dose fall-off affects target coverage in case of positional error.

## Conclusions

Although not dramatic, the increase in target coverage with a shallower gradient in cases of target position error is common for all sized targets under study, and small targets around 1 cm in size had a clearer benefit. In addition, with a shallower gradient, the dose characteristics in the target volume receiving under dose than the prescription showed an additive advantage such that more volumes were irradiated by the nearby dose to the prescription. Therefore, with careful consideration of the increase in 12-Gy volume, the dose gradient can be used as a complementary parameter to reduce coverage deficiency due to targeting error in frameless brain SRS/FSRT.

## Data Availability

Not applicable.

## Abbreviations

SRS: stereotactic radiosurgery
FSRT: fractionated stereotactic radiation treatment
IGRT: image-guided radiotherapy
CBCT: cone beam computed tomography
GI: gradient index
SGRT: surface guided imaging
